# Supplementary material for: Coptis chinensis Franch Directly Inhibits Proteolytic Activation of Kallikrein 5 and Cathelicidin Associated with Rosacea in Epidermal Keratinocytes
Source: Molecules. 2020 Nov 26;25(23):5556. doi: 10.3390/molecules25235556 (PMC7729574; doi:10.3390/molecules25235556)

molecules

MDPI

SUPPLEMENTARY MATERIALS

Article

# *Coptis chinensis* Franch directly inhibits proteolytic activation of kallikrein 5 and cathelicidin associated with rosacea in epidermal keratinocytes.

**Kyung-Baeg Roh [1], Dae-Hoon Ryu [1], Eunae Cho [1], Jin Bae Weon [1], Deokhoon Park [1], Dae-Hyuk Kweon [2] and Eunsun Jung [1,*]**

[1] Biospectrum Life Science Institute, Yongin, 16827, South Korea; biosh@biospectrum.com (K.-B.R); biosc@biospectrum.com (D.-H.R); biozr@biospectrum.com (E.C); biohy@biospectrum.com (J.B.W); pdh@biospectrum.com (D.P); bioso@biospectrum.com (E.J)

[2] Department of Integrative Biotechnology, College of Biotechnology and Bioengineering, Sungkyunkwan University, Suwon, 16419, South Korea; dhkweon@skku.edu (D.-H.K)

* Correspondence: bioso@biospectrum.com; Tel.: +82-70-5117-0029 (E.J.)

## Supplementary materials

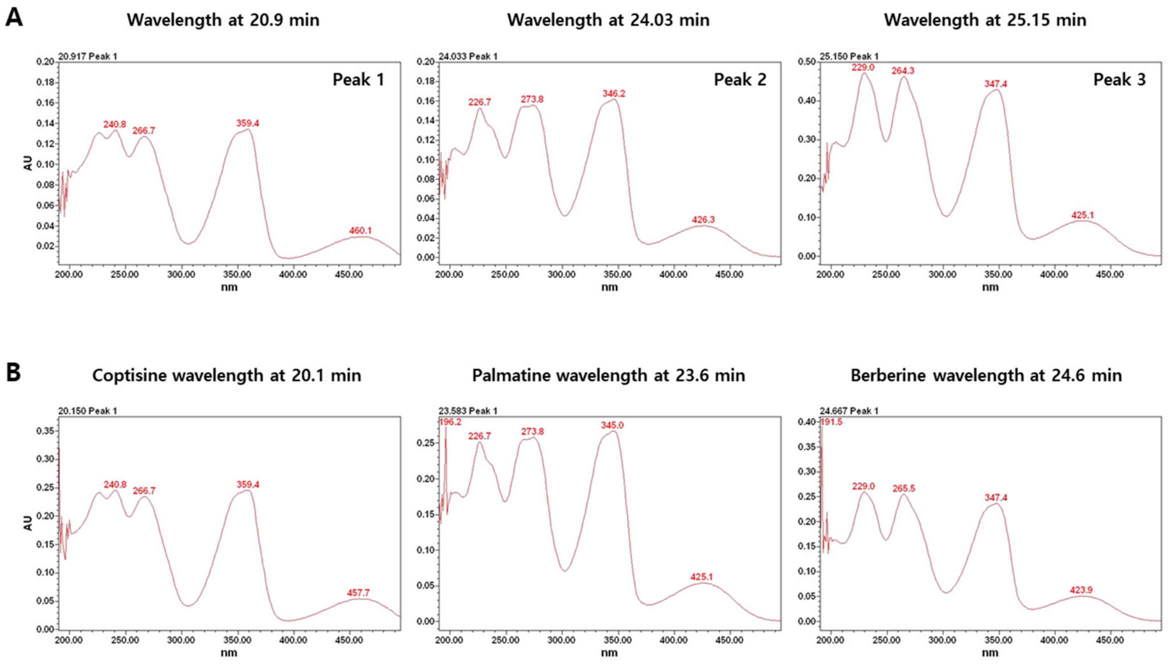


**Figure S1.** Peak identification of CCE (A) by comparing the retention times and UV spectra for individual standards (B).

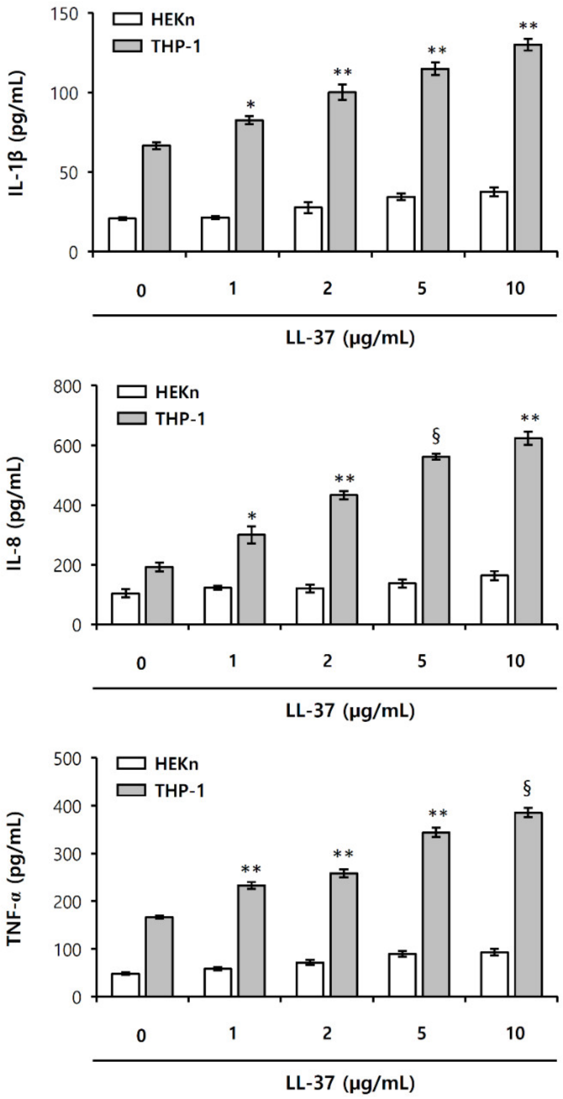


**Figure S2**. LL-37 induces pro-inflammatory cytokines in THP-1. THP-1 cells were seeded in 24-well plates at 2 × $10^5$ cells/well, and differentiated into macrophages using 100 nM PMA for 24 h. The culture media was replaced with fresh DMEM containing 1% FBS and 1% penicillin/streptomycin, and then treated with 1, 2, 5, or 10 μg/mL LL-37 for 48 h. THP-1 culture media was collected, and secreted pro-inflammatory cytokines (IL-1β, IL-8, and TNF-α) were determined by ELISA. HEKn (passage 3) at 90% confluence was treated with 1, 2, 5, or 10 μg/mL LL-37 for 72 h. HEKn culture media was collected, and secreted pro-inflammatory cytokines (IL-1β, IL-8, and TNF-α) were determined by ELISA. **P* < 0.05 *vs*. LL-37-unteated control; ***P* < 0.01 *vs*. LL-37-unteated control; §*P* < 0.001 *vs*. LL-37-unteated control.

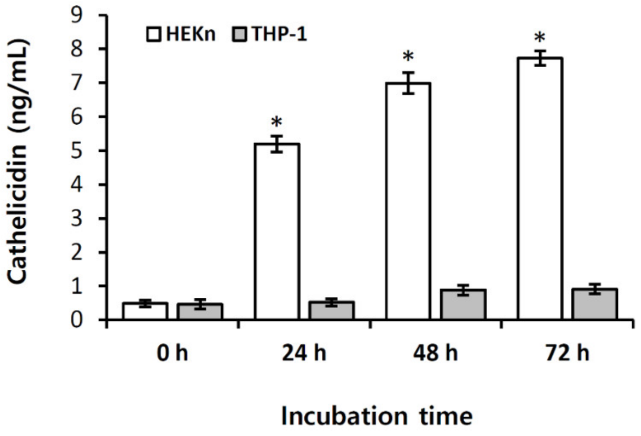


**Figure S3**. VD3 induces cathelicidin expression in HEKn, but not THP-1. HEKn (passage 3) at 90% confluence was treated with 200 nM VD3 for 0, 24, 48, or 72 h. HEKn culture media was collected, and secreted cathelicidins were determined by ELISA. THP-1 cells were seeded in 24-well plates at $2 \times 10^5$ cells/well, and differentiated into macrophages using 100 nM PMA for 24 h. The culture media was replaced with fresh DMEM containing 1 % FBS and 1% penicillin/streptomycin. Subsequently treated with 200 nM VD3 for 0, 24, 48, or 72 h. THP-1 culture media was collected, and secreted cathelicidins were determined by ELISA. *$P < 0.001$ *vs*. 0 h.

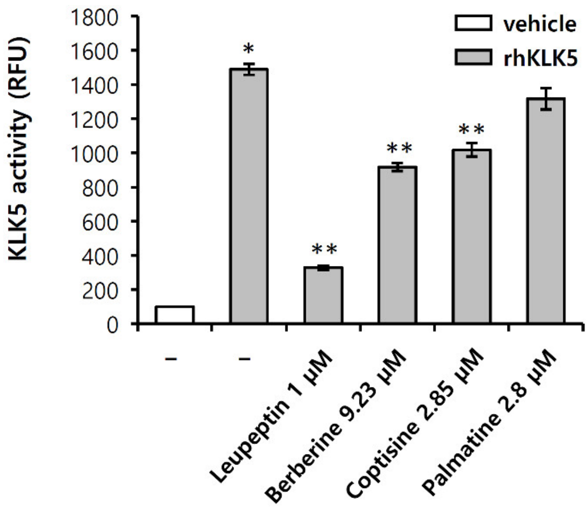


**Figure S4**. The major constituents of CCE inhibits KLK5 protease activity. KLK5 activity was measure in relative fluorescence units (RFU), using fluorogenic peptide substrate sensitive to KLK5. *$P < 0.001$ *vs*. vehicle-treated control; **$P < 0.01$ *vs*. rhKLK5-treated control; DMSO vehicle control (vehicle).

## Full-length Western Blot image of Figure 4

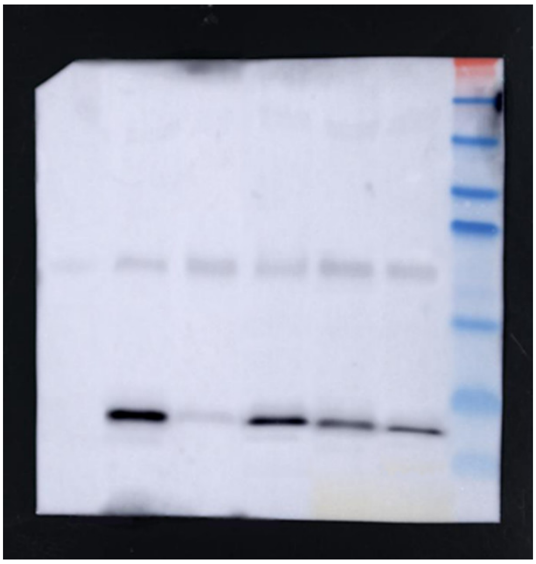

Supplement: Supplementary file 1 [file molecules-25-05556-s001.pdf]
